# Jealousy among Doctors

**Published:** 1886-03

**Authors:** 


### Jealousy Among Doctors.

There is a certain amount of mental force or energy that in nearly every calling can be spent directly in a desire for increase of business. The distiller's conscience doesn't hurt him when he wishes people to drink more whisky. The pastor can legitimately desire the young folk of his flock to marry, so that he can get the fees pertaining to the ceremony. The merchant, without the least self-reproach, can wish that change of fashion or cold weather will make people buy his clothing; and even the lawyer can wish, with all his heart, that rich heirs may contest their father's will, and that, too, with a clear conscience; but no doctor will dare to wish, individually, for people to get sick; he can not find it in his conscience to wish for business except in that general way expressed in the petition, "Give us this day our daily bread;" so he finds employment for the energy which others can put directly into their business, by wishing for his neighbor doctors to starve; or, in other words, that he himself may absorb all the business upon which they collectively depend for a livelihood. Therefore, while it by no means argues perfection, it is really to the credit of the medical profession, in the present state of human weakness, that they exhibit among themselves an unusual degree of jealousy.—*Louisville Medical News.*

